# Supplementary material for: A Comprehensive Assessment of Ultraviolet-Radiation-Induced Mutations in Flammulina filiformis Using Whole-Genome Resequencing
Source: J Fungi (Basel). 2024 Mar 20;10(3):228. doi: 10.3390/jof10030228 (PMC10971301; doi:10.3390/jof10030228)
Supplement: Supplementary file 1 [file jof-10-00228-s001.zip › Supplementary Material S8/KEGG annotation/out/64381550635650.os/KO/out_map/map05120.html]

KEGG PATHWAY: Epithelial cell signaling in Helicobacter pylori infection - Reference pathway


|  |  |
| --- | --- |
| **Epithelial cell signaling in Helicobacter pylori infection - Reference pathway** |  |

[
Pathway menu
| Organism menu
| Pathway entry
| Show description
| User data mapping
]

Two major virulence factors of H. pylori are the vacuolating cytotoxin (VacA) and the cag type-IV secretion system (T4SS) and its translocated effector protein, cytotoxin-associated antigen A (CagA).
VacA binds to lipid rafts and glycosylphosphatidylinositol-anchored proteins (GPI-APs) of the target cell membrane. After insertion into the plasma membrane, VacA channels are endocytosed and eventually reach late endosomal compartments, increasing their permeability to anions with enhancement of the electrogenic vacuolar ATPase (v-ATPase) proton pump. In the presence of weak bases, osmotically active acidotropic ions will accumulate in the endosomes. This leads to water influx and vesicle swelling, an essential step in vacuole formation. In addition, it is reported that the VacA cleavage product binds to the tyrosine phosphatase receptor zeta (Ptprz) on epithelial cells and the induced signaling leads to the phosphorylation of the G protein-coupled receptor kinase-interactor 1 (Git1) and induces ulcerogenesis in mice.
The other virulence factor cag T4SS mediates the translocation of the effector protein CagA, which is subsequently phosphorylated by a Src kinase. Phosphorylated CagA interacts with the protein tyrosine phosphatase SHP-2, thus stimulating its phosphatase activity. Activated SHP-2 is able to induce MAPK signalling through Ras/Raf-dependent and -independent mechanisms. Deregulation of this pathway by CagA may lead to abnormal proliferation and movement of gastric epithelial cells.

|  |  |
| --- | --- |
| Reference pathway | 100% |
